# Acupuncture Alleviates Blood–Brain Barrier Damage After Delayed rtPA Thrombolysis for Acute Ischemic Stroke by Regulating Ferroptosis

**DOI:** 10.1002/brb3.70644

**Published:** 2025-07-10

**Authors:** Zheng Huang, Tianliang Lu, Xinyu Liu, Zhihui Zhang, Yangyang Song, Yiyang Li, Wentao Xu, Xinchang Zhang, Guangxia Ni

**Affiliations:** ^1^ College of Acupuncture‐Moxibustion and Tuina Nanjing University of Chinese Medicine Nanjing China; ^2^ Key Laboratory of Acupuncture and Medicine Research of Ministry of Education, Nanjing University of Chinese Medicine Nanjing China

**Keywords:** acupuncture, acute ischemic stroke, blood–brain barrier, cerebral infarction, ferroptosis, neuron, rtPA thrombolysis

## Abstract

**Background:**

Acute ischemic stroke (AIS) is the second leading cause of death and disability worldwide. Recombinant tissue plasminogen activator (rtPA) was the first FDA‐approved thrombolytic drug for AIS. However, delayed administration of rtPA exacerbates brain injury and increases the risk of hemorrhagic transformation (HT) and mortality. Ferroptosis, an iron‐dependent form of cell death, is closely associated with the pathological process of AIS. Acupuncture, a vital component of traditional Chinese medicine, has been widely used in clinical practice for AIS treatment. This study aims to investigate the protective effects of acupuncture on rats subjected to delayed rtPA thrombolysis in cerebral infarction and its relationship with ferroptosis.

**Methods:**

Adult male Sprague‐Dawley rats were used to establish a thromboembolic stroke model and were randomly assigned to different treatment groups. Xingnao Kaiqiao (XNKQ) acupuncture (at Neiguan and Shuigou acupoints) or sham acupuncture was administered in combination with rtPA thrombolysis. Outcome measures included neurological scores, infarct volume, brain water content, blood–brain barrier (BBB) permeability, expression of iron metabolism‐related proteins, lipid peroxidation levels, and mitochondrial ultrastructural changes.

**Results:**

XNKQ acupuncture significantly improved neurological deficits following delayed rtPA thrombolysis, reduced infarct volume and brain water content, and decreased the incidence of HT and brain edema. By modulating iron metabolism, inhibiting lipid peroxidation, and preserving mitochondrial integrity, acupuncture attenuated neuronal damage and BBB disruption mediated by ferroptosis.

**Conclusion:**

XNKQ acupuncture inhibits neuronal ferroptosis by improving iron metabolism disorders, lipid peroxidation accumulation, and mitochondrial structure, thereby alleviating neuronal damage and BBB disruption, and exerting a protective effect on brain tissue after delayed rtPA thrombolysis.

## Graphical abstract

This study established a rat model of thromboembolic stroke to investigate the effect of Xingnao Kaiqiao acupuncture combined with delayed rtPA thrombolysis. The results showed that acupuncture could alleviate neuronal ferroptosis, improve blood‐brain barrier injury, protect neurological function and reduce the risk of hemorrhagic transformation by regulating iron metabolism, inhibiting lipid peroxidation and protecting mitochondrial structure.

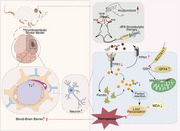

**Abbreviations**
AIS: Acute ischemic stroke
BBB: Blood‐brain barrier
BM: basement membrane
EB: Evans blue
FPN1: Ferroportin 1
GPX4: Glutathione peroxidase 4
GSH: Glutathione
GV26: Shuigou
HT: Hemorrhagic transformation
MCA: Middle cerebral artery
MDA: Malondialdehyde
PC6: Neiguan
rtPA: Recombinant tissue plasminogen activator
TEM: Transmission electron microscopy
TFR1: Transferrin receptor 1
TJs: Tight junctions
XNKQ: Xingnao Kaiqiao

## 1 Introduction

Stroke is the second leading cause of death and disability globally, with acute ischemic stroke (AIS) accounting for 87% of all cases (Feigin et al. 2017). Data from the Global Burden of Disease Study indicate that the age‐standardized incidence of AIS has risen by 34.7% over the past decade (Zeng et al. 2021). The primary treatment for AIS involves restoring cerebral blood flow through intravenous thrombolysis (Lees et al. 2010). Recombinant tissue plasminogen activator (rtPA) was the first recommended intravenous thrombolytic drug approved by the U.S. Food and Drug Administration for clinical use in AIS (S. Liu et al. 2018). Administering rtPA within 4.5 h of symptom onset can yield favorable therapeutic outcomes. However, delayed rtPA treatment may be associated with hemorrhagic transformation (HT) and increased mortality (Yip et al. 2019). Consequently, it is crucial to identify effective adjunct therapies that can be combined with rtPA to reduce the risk of adverse events during delayed rtPA administration.

Ferroptosis is an iron‐dependent form of cell death (Ren et al. 2020) and a key mechanism of pathological cell death in AIS (Aguirre et al. 2005). The Fenton reaction between excess free Fe^2+^ and oxidative respiratory products of mitochondria leads to a significant increase in the depletion of glutathione (GSH) and reactive oxygen species, and inactivation of glutathione peroxidase 4 (GPX4), resulting in an imbalance of the redox system in vivo. Excessive accumulation of the toxic lipid peroxide malondialdehyde (MDA) induces ferroptosis in neurons (Yao et al. 2021). Iron ions play a crucial mediating role in this process (Tuo et al. 2017). In addition, research has indicated that ferroptosis plays a role in the HT that occurs after thrombolysis in the context of cerebral infarction. Mice fed a high‐iron diet exhibit increased levels of lipid peroxidation, which promotes ferroptosis and increases the risk of HT after delayed rtPA thrombolysis (García‐Yébenes et al. 2018).

The blood–brain barrier (BBB) is a biological barrier that separates the central nervous system from the peripheral blood circulation (Zhao et al. 2022), and its structural integrity is largely dependent on tight junctions (TJs). TJs are composed of various proteins, including the claudin family of transmembrane proteins and the ZO family of cytoplasmic attachment proteins. The opening and closing of TJs can modulate BBB permeability. Physiologically, the brain is shielded from systemic iron fluctuations due to the protective function of the BBB. However, during acute ischemia, the BBB is compromised, allowing water, red blood cells, and the iron ions they carry to infiltrate the brain parenchyma. This further promotes ferroptosis, leading to brain edema, HT, and the accumulation of cytotoxic substances. In turn, ferroptosis exacerbates BBB damage, creating a vicious cycle and increasing the risk of thrombolytic complications (W. Wang et al. 2015; Zhou et al. 2021; Arba et al. 2021).

Acupuncture therapy is a crucial component of traditional Chinese medical technology. AIS is a predominant condition within the scope of acupuncture treatment (A. Liu et al. 2016). In clinical practice, the combination of acupuncture therapy with rtPA has emerged as a novel approach for treating AIS. There are numerous acupuncture techniques, each offering distinct therapeutic benefits for different stages of cerebral infarction. Xingnao Kaiqiao (XNKQ) acupuncture is a method developed through extensive clinical trials and was established in 1972 by Professor Xue‐Min Shi, a renowned modern traditional Chinese medicine acupuncturist. The World Federation of Chinese Medicine Societies has disseminated the International Technical Practice of Traditional Chinese Medicine—the treatment of stroke by invigorating the brain and resuscitating acupuncture—to the global community. This acupuncture technique has demonstrated significant therapeutic effects on AIS (Zhuo et al. 2021; Z. Song, Huang, et al. 2022; Z. Zhang et al. 2024; Xinchang et al. 2024).

In our previous experiments, we reported that XNKQ acupuncture treatment mitigated complications such as cerebral hemorrhage resulting from delayed thrombolysis in patients with cerebral infarction (H. Liu et al. 2024). However, the specific mechanisms that account for this relationship have not been clearly defined. In this study, we discovered that XNKQ acupuncture can reduce brain injury following delayed rtPA thrombolysis in cerebral infarction by preserving the integrity of the BBB. Given the strong correlation between complications after delayed rtPA thrombolysis and ferroptosis (García‐Yébenes et al. 2018), we focused on investigating the relationship between ferroptosis and the reduction in BBB damage and neuronal injury following delayed rtPA thrombolysis through XNKQ acupuncture.

## 2 Materials and Methods

### 2.1 Animals

Adult male Sprague‐Dawley rats (300 ± 20 g) and maintained at the SPF level, were obtained from Beijing Vital River Laboratory Animal Technology Co., Ltd. (Beijing, China; License No. SCXK (Jing): 2021‐0006). The rats were maintained in a controlled environment with a humidity of 55 ± 5% and a temperature of 23 ± 3°C under a 12 h light‐dark cycle and were provided with food and water according to a regular schedule. The animal experiments conducted in this study were approved by the Institutional Animal Care and Use Committee of Nanjing University of Chinese Medicine (Approval No. 202404A014).

### 2.2 Animal Grouping

In the first part of the study, the rats were randomly divided into 6 groups: the Sham, Model, rtPA (4.5, 6 h), 6 h rtPA+Acu, and 6 h rtPA+Sham Acu groups. In another part of the study, the rats were randomly divided into 6 groups: the Sham, Model, 6 h rtPA, 6 h rtPA+Acu, 6 h rtPA+Iron‐dextran, and 6 h rtPA+Iron‐dextran+Acu groups. The rats that were injected with the ferroptosis inducer were intraperitoneally injected with Iron‐dextran (0.5g/kg; MedChemExpress, Shanghai, China) 6 h before modeling. Specifically, the model group represented a state of cerebral infarction without rtPA thrombolytic therapy or acupuncture. The sham group did not receive a thrombus injection into the middle cerebral artery (MCA), which was intended to exclude the impact of nonpathological factors during the procedure. The rtPA (4.5, 6 h) group refers to the group receiving rtPA thrombolytic therapy after 4.5 or 6 h of infarction.

### 2.3 Establishment of the Thromboembolic Stroke Model

Blood from the inner canthus of each rat was collected to generate a thrombus, and then the thrombus was transferred to a syringe filled with normal saline. The rats were anesthetized and immobilized, and the blood vessels in the neck were exposed. The syringe containing the thrombus is pushed through the PE‐50 catheter to the beginning of the MCA on the right side, and the MCA blood flow is blocked. The catheter is removed after 5 s (L. Zhang et al. 2015). Laser speckle blood flow imaging (RFLSI ZW, RWD, Shenzhen, China) was employed to monitor cerebral blood flow obstruction and validate the model's effectiveness. A substantial reduction in blood perfusion on the right side indicated successful model construction (H. Wang et al. 2021).

### 2.4 Treatment With Acupuncture and Sham Acupuncture

The rats in the acupuncture group and the sham acupuncture group received corresponding acupuncture treatment or sham acupuncture intervention along with thrombolysis treatment. The acupoints selected were bilateral Neiguan (PC6) and Shuigou (GV26), with the locations of the acupoints referring to the Names and Locations of Common Acupoints in Laboratory Animals Part 2: Rats established by the Chinese Acupuncture Society.

The acupuncture treatment tool used was a stainless steel needle (disposable; diameter: 0.18 mm; depth: 13 mm; Suzhou Hwato Medical Instrument). The acupuncture needles were inserted into points GV26 and PC6, with depths ranging from 2–3 mm. The thumb was used to apply force to the acupuncture needle, resulting in lifting, stabbing, twisting, shrinking, and other techniques, each time it was applied for 1 min, after which the needles were retained for a duration of 30 s.

**FIGURE 1:**
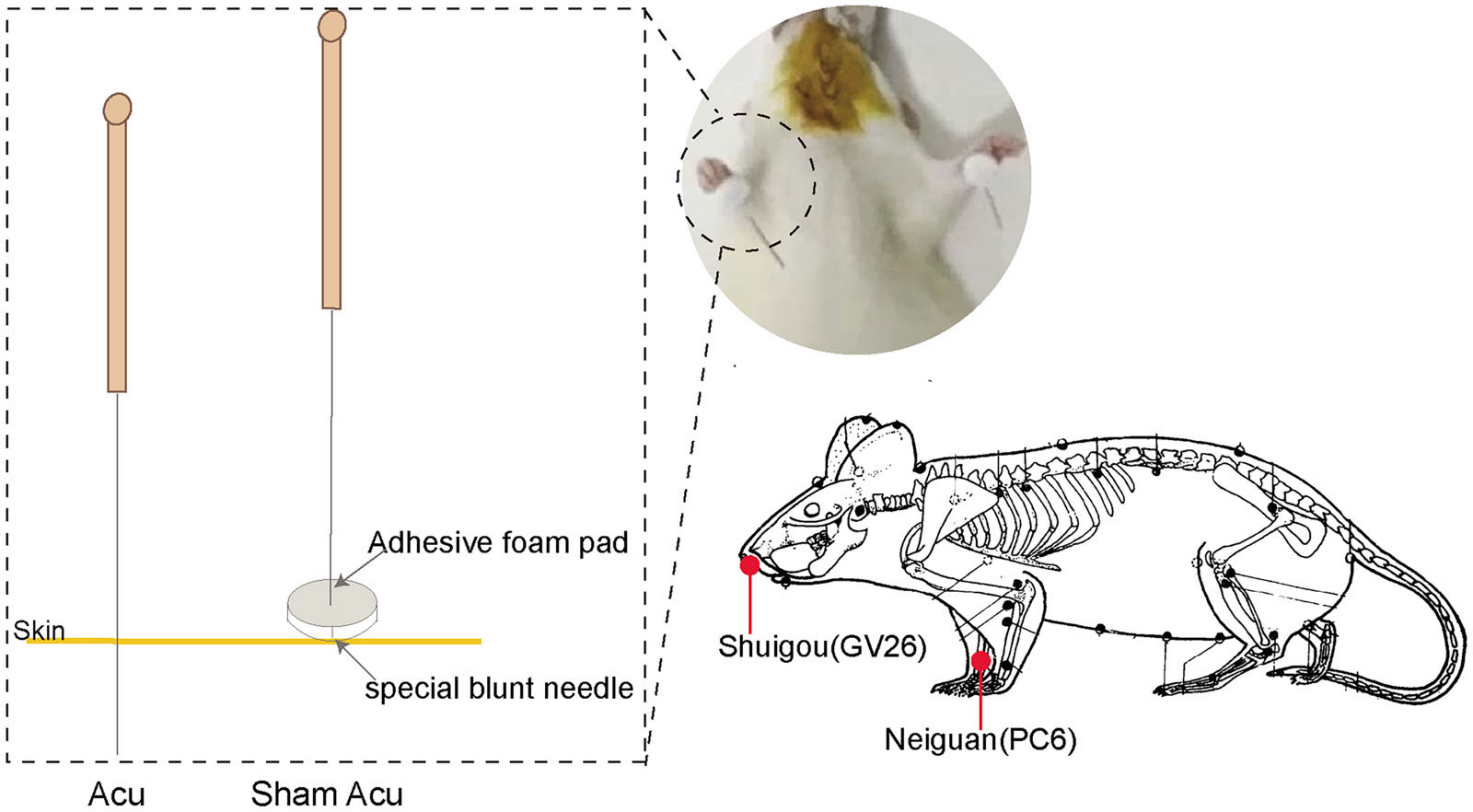
Schematic diagrams of acupuncture (Acu), sham acupuncture (Sham Acu), and acupoint location.

The sham acupuncture scheme was the same as that used in the acupuncture group except that the treatment tools used were different from those used in the acupuncture group. A specialized adhesive foam pad was placed over the GV26 and bilateral PC6 acupoints, and then a specially designed blunt needle was inserted into the foam pad at the designated acupoint location, ensuring that the blunt tip of the needle did not penetrate the skin at the acupoint (Figure 1).

### 2.5 Laser Speckle Flow Imaging

Laser speckle flow imaging (RWD, Shenzhen, China) was used to monitor changes in cerebral ischemic blood perfusion 24 h before surgery and 24 h after surgery. The rats were anesthetized and immobilized, and their skulls were fully exposed. Cranial research was used to sharpen the skull, exposing the right side of the brain. The laser wavelength was set to 785 ± 10 nm, the working height of the laser speckle lens fiber was adjusted to below 10 cm, and the spatial algorithm sliding mode was used, with a spatial filtering constant of 15 s, a frame rate of 0.33 fps, and a recording time of 20 ms. Dynamic perfusion images of blood vessels were collected. The right cerebral cortex blood perfusion (PU) was calculated via RFLSI analysis software.

### 2.6 Assessment of Neurobehavior

After the successful establishment of the model in rats for 24 h, neurological function was assessed via the modified Bederson score (0–5 points), the corner turn test, and the adhesive‐removal test. The modified Bederson score (0–5 points) scoring criteria are as follows: 0, no obvious functional deficits; 1, the rat's left forelimb is mildly flexed; 2, when the tail is pulled, the rat's left forelimb grip strength is significantly reduced, and lifting the rat reveals the left forelimb adhering to the chest wall; 3, the rat's spontaneous activity is nondirectional, and it only turns to the left when the tail is pulled; 4, the rat spontaneously turns to the left and leans to the left when walking; 5, death. In the corner turn test, the rat is placed between a specially designed device consisting of two wooden boards set at a 30° angle. The rat is positioned with its head facing the vertex of the angle. As the rat walks forward and reaches the vertex, it turns at the corner. This process is repeated 10 times, and the test result is indicated by the ratio of the number of pathological turns to the total number of turns. In the adhesive‐removal test, a small piece of adhesive tape is attached to the rat's forelimb, and the time it takes for the rat to remove the tape with its teeth is observed and recorded.

### 2.7 Measurement of the Infarct Volume

Twenty‐four hours after the successful establishment of the rat model, the infarct volume of the brain tissue was measured via TTC staining. The anesthetized rat brain tissue was stored at −20°C for 20 s, after which the brain tissue was cut into 2 mm slices. The brain sections were immersed in 2% triphenyltetrazepine ammonium chloride (TTC; Sigma‐Aldrich, St. Louis, MO, USA) at room temperature and incubated in darkness for 10 s. Image J software (version 1.5.4) was used to analyze the brain slice images. The formula for calculating cerebral infarction volume (%) was as follows: sum of the ischemic area of each section/sum of the brain section area of each section×100%.

### 2.8 Measurement of Brain Water Content

Twenty‐four hours after the successful establishment of the rat model, the wet weight of the brain tissue was measured by weighing. The brain tissue was then placed in an oven at 100°C for 24 h and reweighed to determine the dry weight. The water content (%) was calculated via the following formula: (wet weight‒dry weight)/wet weight×100%.

### 2.9 Assessment of Blood–Brain Barrier (BBB) Permeability

BBB permeability was evaluated via the use of a 2% Evans blue (EB) dye solution (Sigma‐Aldrich, St. Louis, USA). Two hours prior to euthanasia, the rats were administered a tail vein injection of 2% EB dye (0.4 mL/100 g). Twenty‐four hours postsurgery, following systemic perfusion with 0.9% saline, the right brain tissues were excised, weighed, and then submerged in formamide (Macklin, Shanghai, China) at a concentration of 500 µL per 100 mg of tissue for homogenization. The tissues were incubated at 60°C for 24 h, followed by centrifugation for 20 s. The supernatant was harvested, and its absorbance at 620 nm was determined with a spectrophotometer. The concentration of EB (µg/g) was then calculated via the following equation: (EB concentration in µg/mL) × (volume of formamide in mL)/(mass of brain tissue in g).

### 2.10 Measurement of Hemorrhagic Transformation

HT was assessed via a hemoglobin colorimetric assay kit (Beyotime Biotechnology, Shanghai, China). Twenty‐four hours after surgery, brain tissues were extracted from the rats in each group. The side of the right brain tissue was homogenized in 2 mL of phosphate‐buffered saline (PBS). After centrifugation for 30 s, the supernatant was collected, and the absorbance at 410 nm was measured via a spectrophotometer.

### 2.11 Nissl Staining

Nissl staining was utilized to evaluate neuronal injury. Twenty‐four hours after surgery, the rats from each group were anesthetized and perfused systemically with a solution containing 0.9% saline and 4% paraformaldehyde. The brain tissues were subsequently removed and fixed in a paraformaldehyde solution for 24 h. Following dehydration, paraffin sections were prepared. These sections were stained with Nissl staining solution (Solarbio, Beijing, China) for 15 s, and subsequently examined under an optical microscope for any pathological alterations in the brain tissue. The quantity of Nissl‐stained positive cells within the brain sections was quantified via Image J software (version 1.5.4).

### 2.12 Prussian Blue Iron Staining

Iron deposition in the brain tissue was observed via a Prussian blue iron stain kit (enhanced with DAB) (Solarbio, Beijing, China). Twenty‐four hours after surgery, anesthetized rats from each group were perfused systemically with 0.9% saline and 4% paraformaldehyde. The brain tissues were extracted and made into paraffin sections with a thickness of 4 µm. The paraffin sections were stained with Perls working solution at 37°C for 20 s; then, the sections were rinsed with distilled water 3 times, each for 10 s. Stain The sections were incubated with a working solution at 37°C for 20 s and then rinsed with PBS 3 times, each for 10 s. The sections were incubated with an enhanced working solution at 37°C for 20 s, and then rinsed with PBS 3 times, each for 10 s. The samples were stained with a redyeing solution for 5 s and then rinsed with distilled water for 10 s. Iron deposition in the right‐side brain tissues of the rats in each group was observed under an optical microscope, with positive cells containing brown–yellow particles. The area of iron ion (Fe^2+^) deposition in the brain sections was quantified using Image J software.

### 2.13 Ultrastructure by Transmission Electron Microscopy

Transmission electron microscopy (TEM) was used to examine the ultrastructural alterations in the BBB and morphological changes associated with ferroptosis, including changes in the mitochondrial ultrastructure. Following perfusion with PBS and 2.5% glutaraldehyde, the rat brain tissues were promptly excised. Right cortical tissue blocks measuring 1 mm^3^ were placed in a 2.5% glutaraldehyde solution at 4°C and subsequently fixed with a 1% osmium tetroxide solution for 2 h. After undergoing graded dehydration and infiltration with acetone, the tissues were embedded in 618 epoxy resin. Ultrathin sections approximately 90 nm in thickness were prepared via an ultramicrotome and then sequentially stained with uranyl acetate and lead citrate.

### 2.14 Measurement of Brain Iron, Glutathione, and Malondialdehyde

The level of iron ions (Fe^2+^) was detected via an iron assay kit (Dojindo, Kumamoto, Japan). Lipid peroxidation levels were measured with a GSH assay kit and MDA assay kits (Beyotime Biotechnology, Shanghai, China). The right side of the rat cerebral cortex tissue was homogenized and then centrifuged and aliquoted according to the instructions provided with each assay kit. The absorbance of each sample was measured at the corresponding wavelengths (593/405/532 nm) via a spectrophotometer. The contents of Fe^2+^, GSH, and MDA were calculated on the basis of the instructions provided with the assay kits.

### 2.15 Immunofluorescence

The brain tissues were excised and processed into 12 µm‐thick frozen sections. These sections were rinsed three times with PBST and then blocked with PBS containing 0.3% Triton X‐100 and 5% goat serum for 1 h. They were subsequently incubated overnight with primary antibodies, which included Ferritin (1:1000, ab75973, Abcam, Cambridge, UK) and NeuN (1:500, ab104224, Abcam, Cambridge, UK). Afterward, the sections were washed three times with PBST and incubated with an Alexa Fluor 647‐conjugated goat anti‐mouse secondary antibody (1:500, ab150115, Abcam, Cambridge, UK) and an Alexa Fluor 488‐conjugated goat anti‐rabbit IgG antibody (1:500, ab150077, Abcam, Cambridge, UK). Finally, all the sections were mounted with an anti‐fade mounting medium containing DAPI. The images were observed via a fluorescence microscope (Leica Thunder, Wetzlar, Germany) and analyzed via Image J software.

### 2.16 Western Blot Analysis

Proteins were extracted from the right cerebral cortex and quantified via a quantitative BCA assay kit (Beyotime, Shanghai, China). Equal amounts of protein were resolved by 10% SDS‒PAGE and transferred to PVDF membranes (Millipore, Burlington, MA, USA). The membranes were incubated overnight at 4°C with the following primary antibodies: Claudin5 (1:1000, 29767‐1‐AP, Proteintech, Wuhan, China), ZO‐1 (1:1000, 21773‐1‐AP, Proteintech, Wuhan, China), Ferritin (1:1000, ab75973, Abcam, Cambridge, UK), GPX4 (1:1000, ab125066, Abcam, Cambridge, UK), FPN1 (1:500, DF13561, Affinity, Jiangsu, China), TFR1 (1:500, AF5343, Affinity, Jiangsu, China), Solute Carrier Family 7 Member 11 (SLC7A11) (1:500, DF12509, Proteintech, Jiangsu, China), and GAPDH (1:2000, UM4002, Utibody, Tianjin, China). The samples were then incubated for 1 h at room temperature with horseradish peroxidase‐conjugated goat anti‐rabbit IgG(H+L) or goat anti‐mouse IgG(H+L) (1:10,000, Affinity, Jiangsu, China). Following chemiluminescence detection with ECL reagents and a Fusion Edge Multi‐Function Imaging System (Vilber, Collégien, France), the relative optical densities of the protein bands were quantified via Image J software.

### 2.17 Statistical Analysis

The data were processed via SPSS software (version 23.0) and are expressed as the mean±standard deviations. The Shapiro‒Wilk normality test was used to examine whether the data were normally distributed. When the data were normally distributed and had equal variances, differences among multiple groups were analyzed via one‐way ANOVA, with post‐hoc multiple comparisons performed via the least significant difference (LSD) test for pairwise comparisons between groups. If the data did not conform to a normal distribution, nonparametric tests were used for analysis. If the data had unequal variances, Dunn's test was applied. GraphPad software (version 9.5.0) was used to create statistical graphs. A difference was considered statistically significant if p < 0.05.

## 3 Results

### 3.1 Effect of Acupuncture on Brain Injury After Delayed rtPA Thrombolysis in Rats With Cerebral Infarction

**FIGURE 2:**
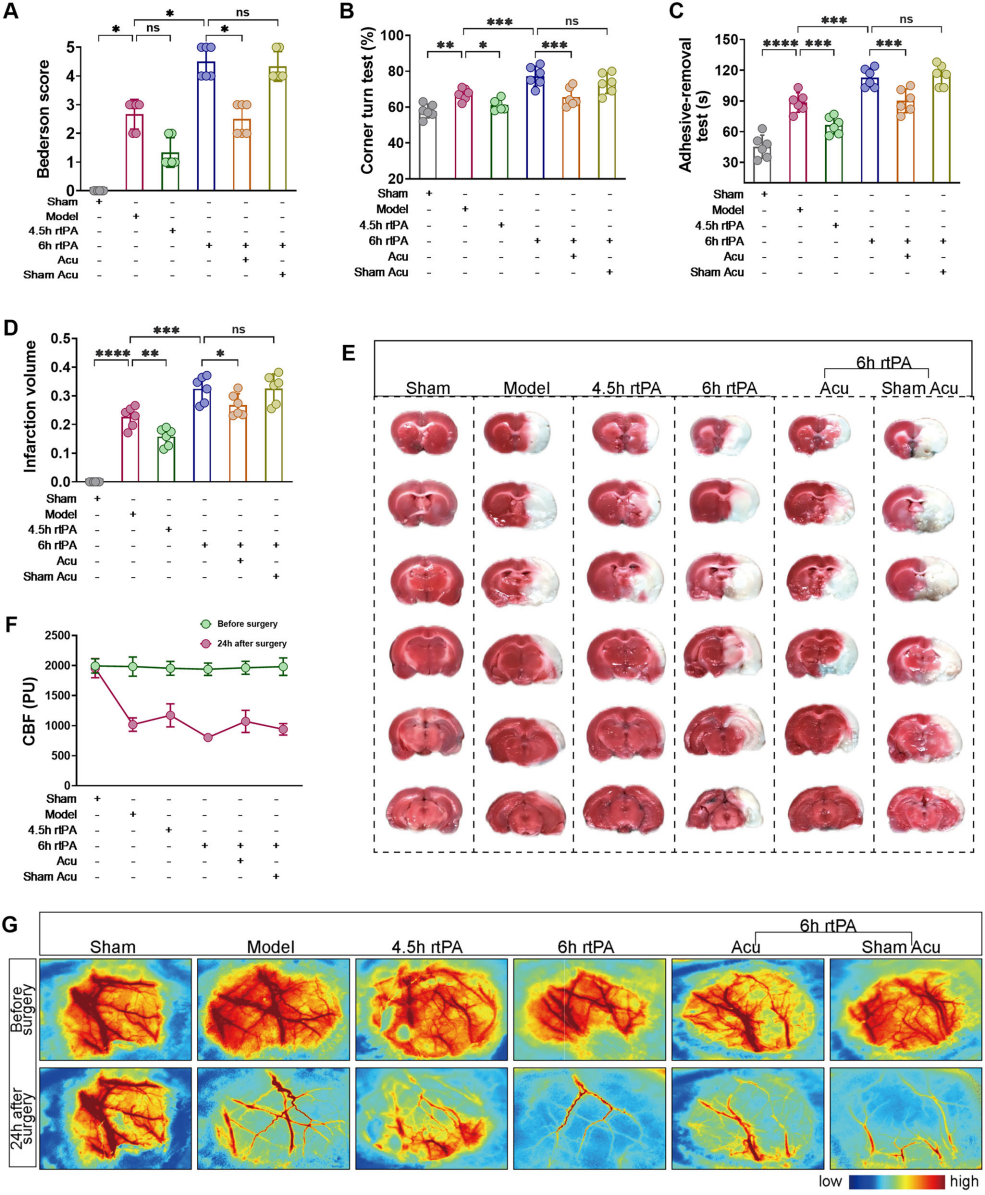
Acupuncture alleviates brain damage following delayed rtPA thrombolysis in rats with cerebral infarction. (**A–C**) Neurological deficits were assessed in each group of rats via the Bederson score, corner turn test, and adhesive‐removal test (*n* = 6). (**D,E**) TTC staining was used to determine the infarct volume ratio and representative images of brain slices in each group of rats (*n* = 6). (**F,G**) Blood flow perfusion in the affected cerebral cortex and representative images were measured in each group of rats. * denotes *p* < 0.05, ** denotes *p* < 0.01, *** denotes *p* < 0.001.

To verify the neuroprotective effect of acupuncture after delayed rtPA thrombolysis in rats with cerebral infarction, we first assessed neurological function via the Bederson score, corner turn test, and adhesive‐removal test. The results shown in Figure 2A–C indicate that compared with the Model group, the rats treated with 4.5 h of rtPA thrombolysis presented lower Bederson scores, fewer corner turns, and shorter adhesive‐removal times. In contrast, the rats treated with 6 h of rtPA thrombolysis presented significantly higher Bederson scores, greater corner turn counts, and longer adhesive‐removal times, suggesting that thrombolysis within the 4.5 h time window can salvage neurological function in rats, whereas delayed thrombolysis outside this window at 6 h can lead to severe neurological deficits. Compared with those in the 6 h rtPA group, the Bederson scores, corner turn counts, and adhesive‐removal times of the rats in the 6 h rtPA+Acu group tended to normalize, whereas those in the 6 h rtPA+Sham Acu group tended to change little, indicating that acupuncture can mitigate the neurological deficits caused by 6 h delayed rtPA thrombolysis in cerebral infarction.

TTC staining and laser speckle flow imaging were used to observe cerebral infarct volume and blood flow perfusion. As shown in Figure 2D–G, compared with the Model group, the rats in the 4.5 h rtPA group presented reduced infarct volumes and increased blood flow perfusion in the affected cerebral cortex, whereas the rats in the 6 h rtPA group presented increased infarct volumes and significantly lower blood flow perfusion in the affected cerebral cortex. This finding indicates that after cerebral infarction, the infarct volume in the rats did not significantly expand under thrombolysis treatment within the 4.5 h time window and that blood flow perfusion was restored. However, delayed thrombolysis outside this window at 6 h led to a significant increase in infarct volume and blood flow perfusion. Compared with those in the 6 h rtPA group, the infarct volume and blood flow perfusion in the affected cerebral cortex were lower in the 6 h rtPA+Acu group, whereas the infarct volume and blood flow perfusion in the 6 h rtPA+Sham Acu group were not significantly different. These findings suggest that acupuncture can salvage the increased infarct volume and blood flow perfusion caused by delayed rtPA thrombolysis.

Overall, these data indicate that acupuncture significantly mitigates neurological deficits, infarct volume, and blood flow perfusion caused by delayed rtPA thrombolysis in rats with cerebral infarction.

### 3.2 Influence of Acupuncture on Complications Caused by Delayed rtPA Thrombolysis in Rats With Cerebral Infarction

**FIGURE 3:**
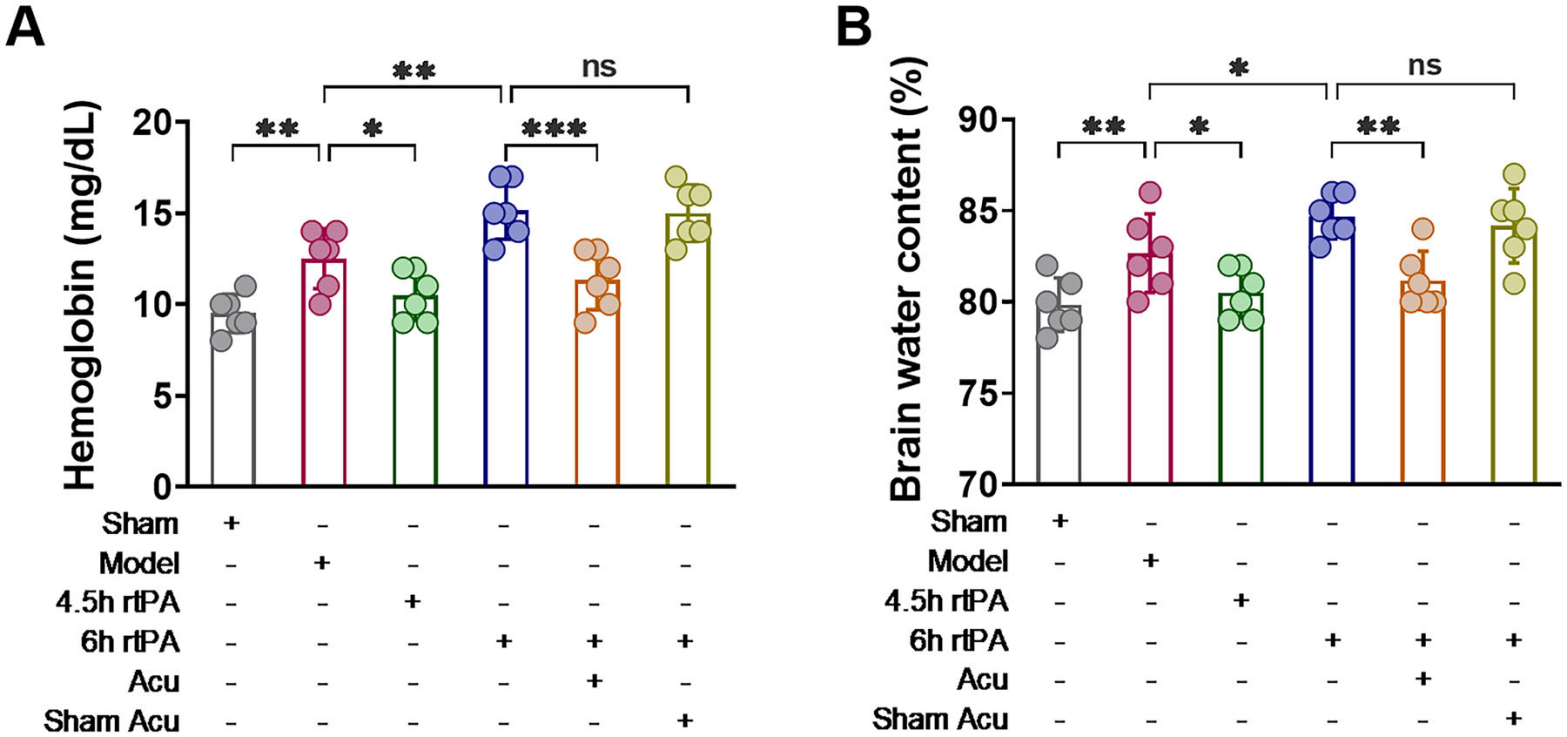
Acupuncture mitigates complications caused by delayed rtPA thrombolysis in rats with cerebral infarction. (**A**) Hemoglobin content in the affected cerebral cortex of each group of rats (*n* = 6). (**B**) Water content in the affected brain tissue of each group of rats (*n* = 6). * denotes *p* < 0.05, ** denotes *p* < 0.01, *** denotes *p* < 0.001.

Cerebral infarction with delayed rtPA thrombolysis is prone to complications such as intracranial hemorrhage and brain edema. A hemoglobin quantitative detection kit was used to assess intracranial hemorrhage. The results shown in Figure 3 indicate that we evaluated the degree of brain edema through brain water content detection. As shown in Figure 3, compared with the Model group, the 6 h rtPA group presented increased hemoglobin and brain water contents. Compared with those in the 6 h rtPA group, there was a notable reduction in both hemoglobin and brain water content in the 6 h rtPA+Acu group, whereas the 6 h rtPA+Sham Acu group presented no significant alterations. These findings suggest that delayed rtPA thrombolysis in cerebral infarction rats can cause severe intracranial hemorrhage and brain edema, whereas acupuncture treatment can improve the degree of bleeding and edema. These findings indicate that acupuncture can reduce complications such as intracranial hemorrhage and brain edema caused by delayed rtPA thrombolysis in rats with cerebral infarction.

### 3.3 Influence of Acupuncture on the Blood‒Brain Barrier After Delayed rtPA Thrombolysis in Cerebral Infarction Rats

**FIGURE 4:**
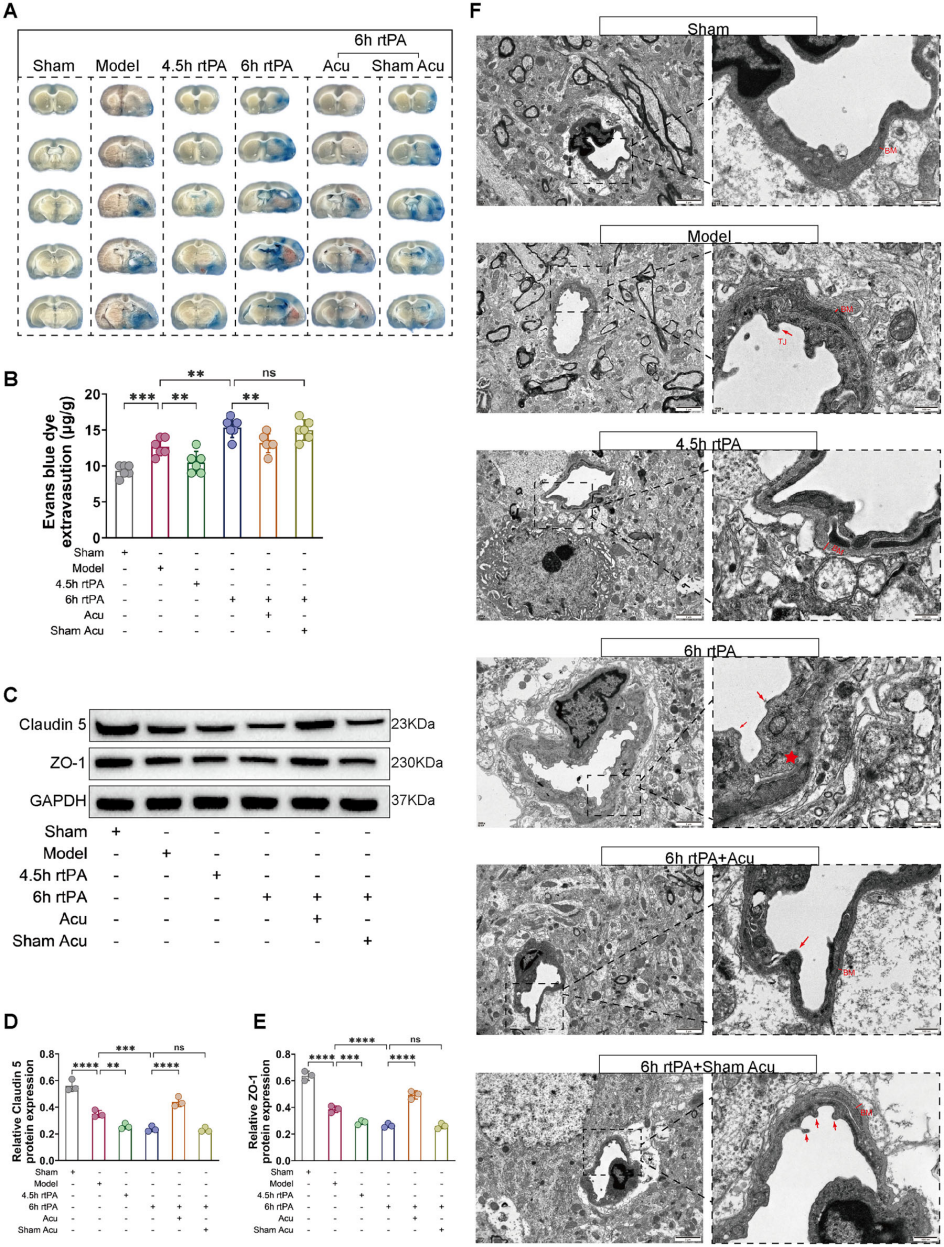
Acupuncture alleviates BBB disruption following delayed rtPA thrombolysis in rats with cerebral infarction. (**A,B**) Representative images and EB dye extravasation in each group of rats (*n* = 6). (**C–E**) Representative Western blot images and quantification of the expression of the TJ‐associated proteins Claudin 5 and ZO‐1 in the affected cerebral cortex of the rats in each group (*n* = 3). (**F**) Representative TEM images of the BBB of the affected brain tissue in each group of rats (*n* = 3) (left, scale bar = 2 µm; right, scale bar = 500 nm). Red arrow indicates the position of TJ (TJ, tight junction), red straight line refers to the thickness of BM (BM, basement membrane), red asterisks indicate severe swelling and deformation in this region. The representative image captures key pathological hallmarks. * denotes *p* < 0.05, ** denotes *p* < 0.01, *** denotes *p* < 0.001.

BBB disruption is a direct cause of complications such as HT and brain edema following thrombolysis (Jiang et al. 2018; Dharmasaroja 2016), and its normal function is closely related to permeability and structural integrity. First, EB staining was used to observe changes in the BBB permeability. As shown in Figure 4A–B, EB dye leaked into the brain parenchyma through the BBB in the brain tissue of the rats in the Model group. Compared with the Model group, the 4.5 h rtPA group had less EB leakage, but the 6 h rtPA group had significantly more EB leakage. These findings indicate that delayed rtPA thrombolysis increases BBB permeability. Compared with the 6 h rtPA group, the 6 h rtPA+Acu group presented a significant reduction in EB leakage, whereas the 6 h rtPA+Sham Acu group did not significantly decrease EB leakage. These findings suggest that acupuncture can significantly reduce EB leakage in the brain tissue of rats subjected to delayed rtPA thrombolysis, thereby improving BBB permeability.

Second, we observed changes in the expression of the TJs proteins Claudin5 and ZO‐1 to understand the effects of acupuncture on the structural integrity of the BBB after delayed rtPA thrombolysis. As shown in Figure 4C–E, compared with those in the Model group, the protein levels of Claudin5 and ZO‐1 were lower in the 6 h rtPA group, indicating that delayed rtPA thrombolysis exacerbates the abnormal expression of TJ‐related proteins, leading to disruption of BBB structural integrity. Compared with those in the 6 h rtPA group, there was a marked increase in the protein expression levels of Claudin5 and ZO‐1 in the 6 h rtPA+Acu group, whereas the levels in the 6 h rtPA+Sham Acu group were not significantly different. These results indicate that acupuncture can regulate the expression of TJ‐related proteins after delayed rtPA thrombolysis, modulating the opening and closing of TJs and thereby improving BBB integrity.

Third, we observed changes in the ultrastructures of TJs in the BBB via TEM. As shown in Figure 4F, the morphology of the BBB in the sham group was normal, with a smooth and intact basement membrane (BM) surface and no swelling observed, and the structure of the TJs was dense and complete. In the Model group, the endothelial cells and BM were swollen and deformed, the connections between the endothelial cell layers were loose, and the TJs were damaged. In the 6 h rtPA group, the swelling and deformation of the endothelial cells and BM in the BBB were further exacerbated, the TJs were disrupted, and the ultrastructure of the BBB was severely damaged. In the 6 h rtPA+Acu group, the degree of ultrastructural damage to the BBB was reduced, with less swelling of the endothelial cells and BM, the morphology was essentially intact, and the disruption of the TJs was significantly reduced. The disruption of TJs in the 6 h rtPA+Sham Acu group did not significantly improve.

In summary, after delayed rtPA thrombolysis in cerebral infarction, EB leakage, increased BBB permeability, abnormal expression of TJ‐related proteins, and severe morphological damage to TJs increase, leading to significant disruption of BBB structural integrity. However, acupuncture treatment can significantly reduce EB leakage in the brain tissue of rats with delayed rtPA thrombolysis, improve BBB permeability, regulate the expression of TJ‐related proteins, alleviate morphological damage to TJs, and maintain the integrity of the BBB structure.

### 3.4 Effect of Acupuncture on Neurons After Delayed rtPA Thrombolysis in Rats With Cerebral Infarction

**FIGURE 5:**
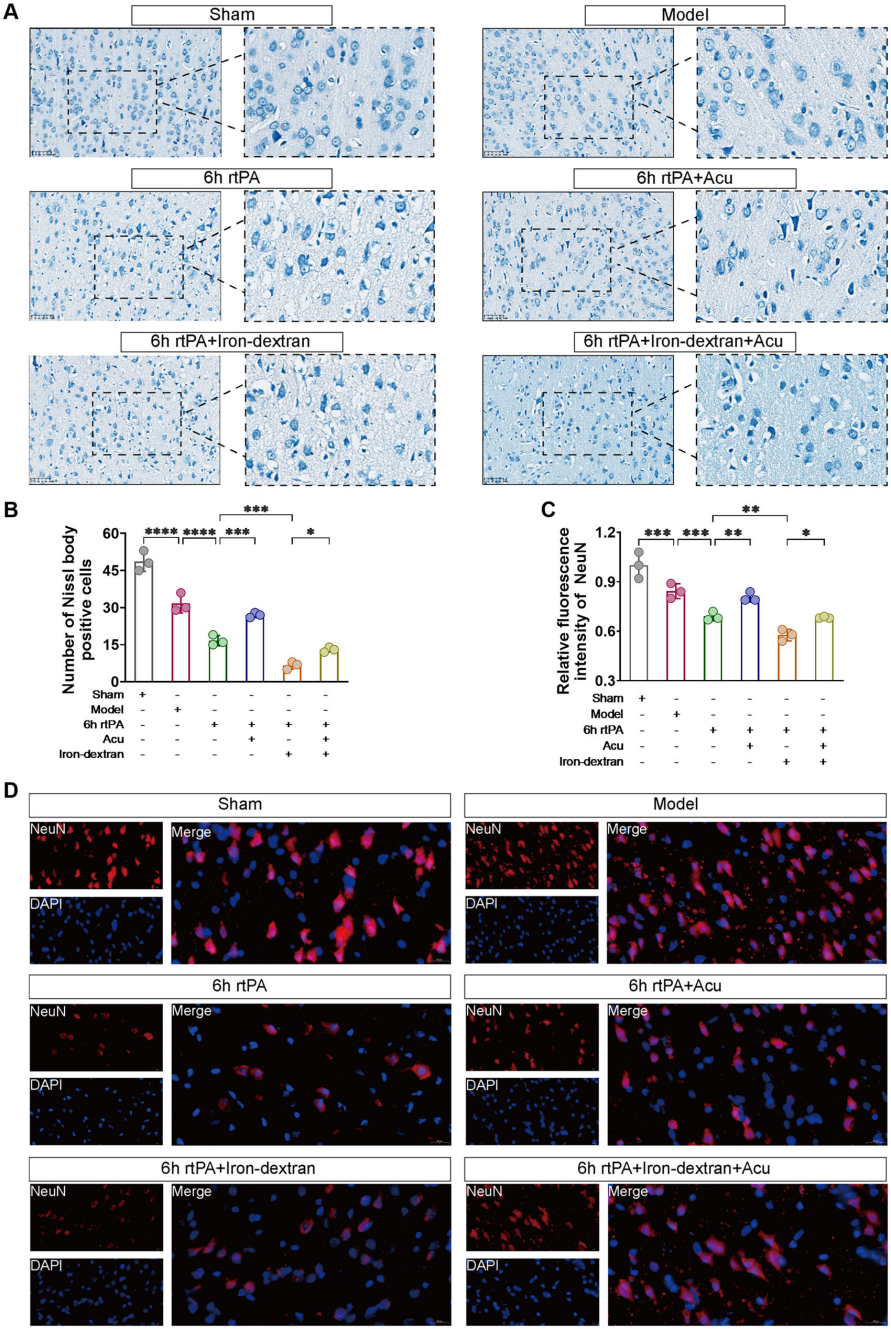
Acupuncture mitigates neuronal damage following delayed rtPA thrombolysis in rats with cerebral infarction. (**A**) Representative images of Nissl staining of the affected brain tissue of each group of rats (scale bar, 50 µm) and (**B**) the number of Nissl‐positive neurons (*n* = 3). (**C**) Relative fluorescence intensity of NeuN in the affected brain tissue of each group of rats (*n* = 3). (**D**) Representative images of NeuN (red) immunofluorescence staining (scale bar, 20 µm). * denotes *p* < 0.05, ** denotes *p* < 0.01, *** denotes *p* < 0.001.

After cerebral infarction, the BBB is disrupted, and neurons are damaged. We first used Nissl staining to observe the extent of neuronal damage. As shown in Figure 5A,B, compared with those in the Model group, the neurons in the 6 h rtPA group presented a shrunken morphology, fewer Nissl bodies, and blurred contours, indicating that delayed rtPA thrombolysis in cerebral infarction led to morphological damage to neurons, a decrease in Nissl bodies, and increased neuronal damage. Subsequently, we artificially exacerbated ferroptosis using an exogenous ferroptosis inducer (Iron‐dextran) to verify whether delayed thrombolysis‐induced neuronal damage is mediated through the ferroptosis pathway. Compared with those in the 6 h rtPA group, the neurons in the 6 h rtPA+Iron‐dextran group presented severe morphological damage, a further reduction in Nissl bodies, and the disappearance of contours. After acupuncture treatment, compared with both the 6 h rtPA and 6 h rtPA+Iron‐dextran groups, the morphology of neurons in both the 6 h rtPA+Acu group and the 6 h rtPA+Iron‐dextran+Acu group improved, and the number of Nissl body‐positive cells increased accordingly, indicating that acupuncture intervention may improve neuronal morphology and increase the number of Nissl body‐positive cells by regulating ferroptosis‐related pathways.

Next, we used immunofluorescence staining to observe neuronal damage, marking neurons with the neuron‐specific nuclear protein NeuN. As shown in Figure 5C,D, compared with that in the Model group, the relative fluorescence intensity of NeuN was lower in the 6 h rtPA group, indicating that delayed rtPA thrombolysis in cerebral infarction causes neuronal damage. After iron‐induced ferroptosis, the relative fluorescence intensity of NeuN was even lower in the 6 h rtPA+Iron‐dextran group than in the 6 h rtPA group. After acupuncture intervention, compared with both the 6 h rtPA and 6 h rtPA+Iron‐dextran groups, the relative fluorescence intensity of NeuN increased in both the 6 h rtPA+Acu group and the 6 h rtPA+Iron‐dextran+Acu group, indicating that acupuncture intervention may alleviate neuronal damage by modulating ferroptosis‐related pathways.

### 3.5 Influence of Acupuncture on Iron Metabolism After Delayed rtPA Thrombolysis in Rats With Cerebral Infarction

**FIGURE 6:**
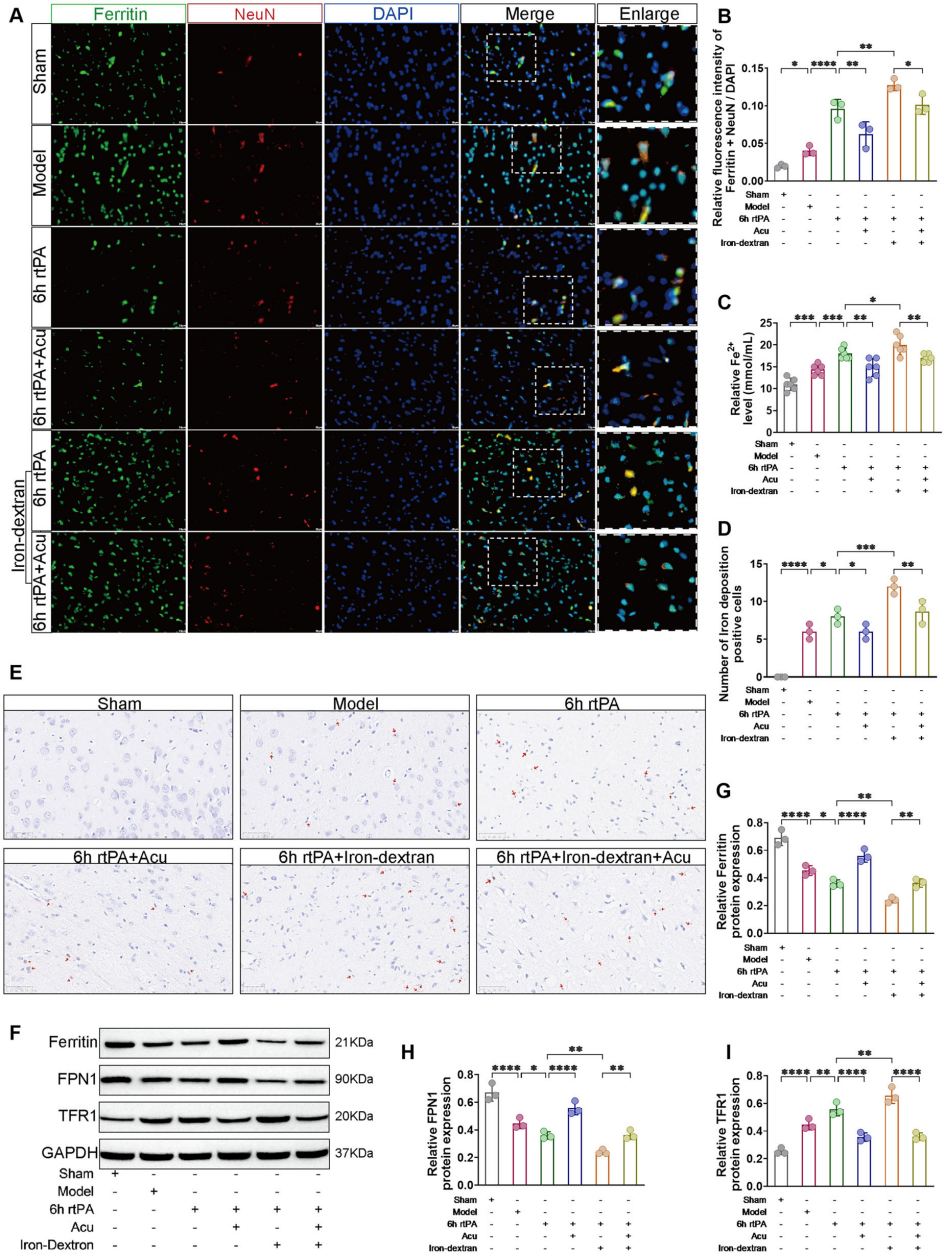
Acupuncture regulates iron metabolism disorders following delayed rtPA thrombolysis in rats with cerebral infarction. (**A**) Representative immunofluorescence images of Ferritin (green) increase in NeuN (red) (scale bar = 20 µm). (**B**) Bar graphs showing quantitative evaluation of Ferritin increase in neurons (*n* = 3). (**C**) Detection of Fe^2+^ content in the affected cerebral cortex of each group of rats (*n* = 6). (**D**) Positive cell counts of iron deposition observed by Prussian blue staining in the affected brain tissue of each group of rats (red arrows indicate brown granules representing Fe^2+^ deposition) (*n* = 3) and (**E**) representative Prussian blue staining images (scale bar = 50 µm). (**F–I**) Representative Western blot images and protein expression levels of the iron metabolism‐related proteins Ferritin, FPN1, and TFR1 in the affected cerebral cortex of each group of rats (*n* = 3). * denotes *p* < 0.05, ** denotes *p* < 0.01, *** denotes *p* < 0.001.

Iron metabolism disorder is a typical characteristic that distinguishes ferroptosis from other forms of programmed cell death. To investigate the changes in iron metabolism in neurons following delayed rtPA thrombolysis in cerebral infarction, we first used immunofluorescence colocalization to visually observe the distribution and changes in Ferritin in NeuN. As shown in Figure 6A,B, compared with those in the Model group, the colocalization levels of NeuN (red) and Ferritin (green) were greater in the 6 h rtPA group; compared with those in the 6 h rtPA group, the colocalization levels of NeuN and Ferritin were even greater in the 6 h rtPA+Iron‐dextran group. These findings indicate that Ferritin is expressed and accumulates in neurons after delayed rtPA thrombolysis in cerebral infarction and that the degradation of Ferritin may exacerbate neuronal damage. After acupuncture treatment, compared with both the 6 h rtPA and 6 h rtPA+Iron‐dextran groups, the colocalization levels of NeuN and Ferritin were correspondingly reduced in both the 6 h rtPA+Acu group and the 6 h rtPA+Iron‐dextran+Acu group. These findings suggest that acupuncture may exert neuroprotective effects by regulating iron metabolism processes and inhibiting excessive neuronal ferritin deposition, potentially through suppression of ferroptosis pathways.

Next, we used Fe^2+^ content detection and Prussian blue staining to observe the degree of iron deposition. As shown in Figure 6C–E, compared with the 6 h rtPA group, the 6 h rtPA+Iron‐dextran group presented increased Fe^2+^ content and a greater number of brown granules (representing Fe^2+^ deposition), indicating that delayed rtPA thrombolysis in cerebral infarction leads to the accumulation of free Fe^2+^ and more severe iron deposition. The severe accumulation of free Fe^2+^ may be related to ferroptosis. After acupuncture treatment, compared with both the 6 h rtPA and 6 h rtPA+Iron‐dextran groups, the accumulation of free Fe^2+^ and the number of brown granules were reduced in both the 6 h rtPA+Acu group and the 6 h rtPA+Iron‐dextran+Acu group, indicating that acupuncture can alleviate the accumulation of free Fe^2+^ caused by delayed rtPA thrombolysis and reduce iron deposition.

Western blot analysis was employed to quantify the expression levels of proteins involved in iron metabolism, specifically Ferritin, Ferroportin 1 (FPN1), and Transferrin Receptor 1 (TFR1). As depicted in Figure 6F–I, compared with that in the 6 h rtPA group, the expression of Ferritin and FPN1 was significantly lower, whereas TFR1 expression was notably elevated in the 6 h rtPA+Iron‐dextran group. Following acupuncture treatment, compared with both the 6 h rtPA and 6 h rtPA+Iron‐dextran groups, the expression levels of Ferritin and FPN1 were restored, and TFR1 expression was reduced in both the 6 h rtPA+Acu group and the 6 h rtPA+Iron‐dextran group. These findings indicate that acupuncture effectively modulates the aberrant expression of iron metabolism‐related proteins following delayed rtPA thrombolysis for 6 h in rats with cerebral infarction.

Overall, following delayed rtPA thrombolysis in cerebral ischemia, there is an accumulation of Ferritin in neurons, increased degradation of ferritin‐releasing Fe^2+^, abnormal expression of iron metabolism‐related proteins, and disordered iron ion metabolism, with free Fe^2+^ accumulation in neurons and severe iron deposition. However, acupuncture treatment can regulate the abnormal expression of iron metabolism‐related proteins, reduce iron deposition in neurons, and maintain iron metabolism levels, thereby inhibiting ferroptosis and reducing neuronal damage.

### 3.6 Effect of Acupuncture on Lipid Peroxidation Levels After Delayed rtPA Thrombolysis in Rats With Cerebral Infarction

**FIGURE 7:**
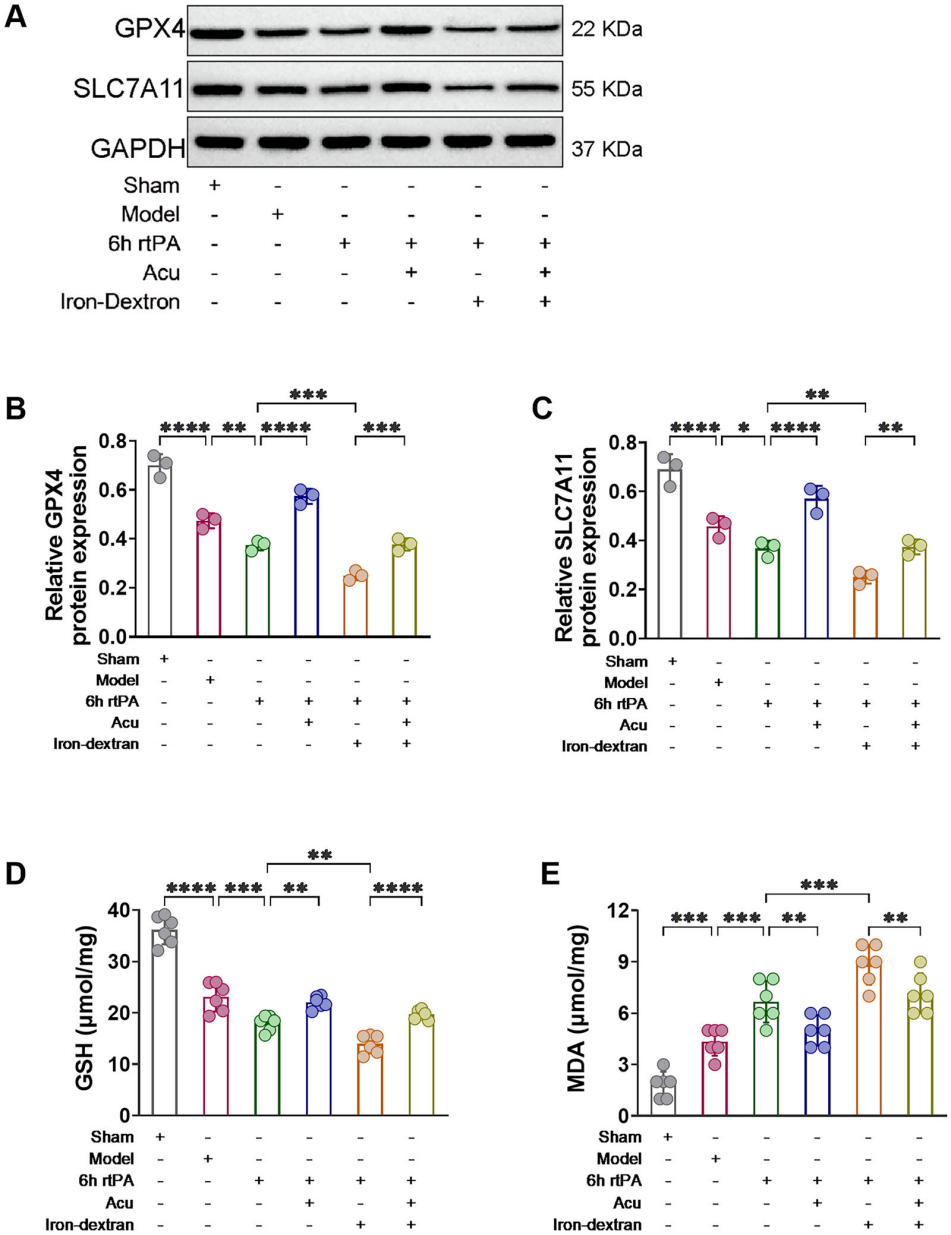
Acupuncture mitigates lipid peroxidation levels following delayed rtPA thrombolysis in rats with cerebral infarction. (**A–C**) Representative Western blot images and protein expression of the lipid peroxidation‐related proteins GPX4 and SLC7A11 in the affected cerebral cortex of each group of rats (*n* = 3). (**D**) Detection of GSH levels in the affected cerebral cortex of each group of rats (*n* = 6). (**E**) Detection of MDA levels in the affected cerebral cortex of each group of rats (*n* = 6). * denotes *p* < 0.05, ** denotes *p* < 0.01, *** denotes *p* < 0.001.

Evaluating lipid peroxidation levels is a hallmark feature that differentiates ferroptosis from other modes of programmed cell death (Kenny et al. 2019). Western blot analysis was used to quantify the expression levels of the antioxidant‐associated proteins SLC7A11 and GPX4, while the content of GSH and the lipid peroxidation product MDA were assessed to determine the extent of lipid peroxidation. As shown in Figure 7, at 6 h after rtPA thrombolysis in cerebral infarction, the levels of the negative regulators GSH, GPX4, and SLC7A11 were reduced, and the level of the lipid peroxidation product MDA was increased. These changes were exacerbated by Iron‐dextran‐induced ferroptosis. However, after acupuncture treatment, compared with both the 6 h rtPA and 6 h rtPA+Iron‐dextran groups, the levels of the negative regulators GSH, GPX4, and SLC7A11 were significantly increased, and the level of the lipid peroxidation product MDA was decreased in both the 6 h rtPA+Acu group and the 6 h rtPA+Iron‐dextran+Acu group. These findings demonstrate that acupuncture can effectively counteract the antioxidant defense system impairment induced by delayed rtPA thrombolysis and ferroptosis. By upregulating key antioxidant proteins (GPX4 and SLC7A11), restoring GSH content, and reducing MDA levels, acupuncture successfully reestablishes cellular redox homeostasis. This confirms that acupuncture alleviates neuronal damage following delayed thrombolysis in cerebral infarction by modulating ferroptosis‐associated lipid peroxidation pathways, thereby providing molecular‐level mechanistic insights into its neuroprotective effects.

### 3.7 Changes in the Mitochondrial Structure of Ferroptosis After Delayed rtPA Thrombolysis in Rats With Cerebral Infarction Induced by Acupuncture

**FIGURE 8:**
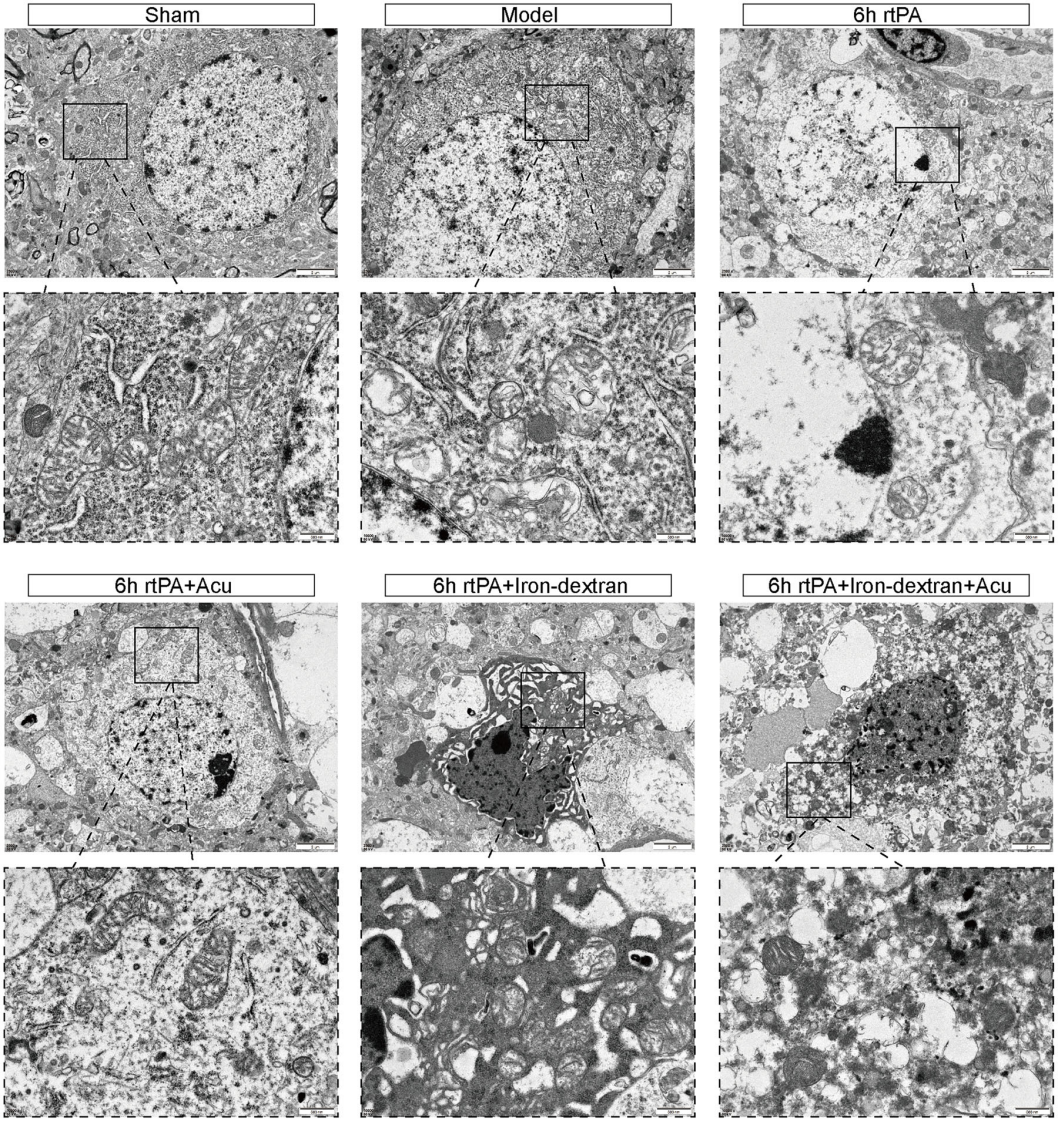
Representative TEM images of the mitochondrial ultrastructure during ferroptosis in each group of rats (*n* = 3) (left, scale bar = 2 µm; right, scale bar = 500 nm).

Mitochondria are important intracellular storage sites for iron. Ferroptosis is characterized by the destruction of the mitochondrial structure at the morphological level (Y. Zhang et al. 2021). We observed the ultrastructure of mitochondria in neurons via TEM. In the Sham group, the double membrane and cristae of the mitochondria were intact, with no abnormalities. In the Model group, the mitochondria were shrunken, with increased membrane density, reduced cristae, and ruptured outer membranes. In the 6 h rtPA group, the mitochondria further shrank, with significantly reduced or absent cristae, vacuolization, and outer membrane rupture. Compared with those in the 6 h rtPA group, the mitochondrial structure and shape in the 6 h rtPA+Acu group were essentially normal, with visible cristae. Damage to the cells in the 6 h rtPA+Iron‐dextran group was further exacerbated, with severe mitochondrial shrinkage, even fewer cristae, and increased rupture of the outer membranes. Compared with that in the 6 h rtPA+Iron‐dextran group, the mitochondrial morphology in the 6 h rtPA+Iron‐dextran+Acu group was improved (Figure 8). These results indicate that delayed rtPA thrombolysis post‐cerebral infarction induces characteristic ferroptosis‐associated mitochondrial damage including ultrastructural alterations such as shrinkage, cristae reduction, and outer membrane rupture. Acupuncture treatment significantly ameliorates these pathological changes, not only restoring near‐normal mitochondrial morphology in the 6 h rtPA group but also reversing Iron‐dextran‐aggravated mitochondrial injury. This confirms that acupuncture counteracts ferroptosis progression by preserving mitochondrial structural and functional integrity, providing direct ultrastructural evidence for its neuroprotective effects against neuronal damage.

In summary, delayed rtPA thrombolysis after cerebral infarction can disrupt the BBB and damage neurons. This leads to changes in neuronal morphology, a reduction in the number of Nissl bodies, and a decrease in the relative fluorescence intensity of NeuN. Meanwhile, it triggers iron metabolism disorders, manifested as the accumulation of Ferritin, an increase in free Fe^2+^, and abnormal expression of iron metabolism‐related proteins. It also causes an elevation in the level of lipid peroxidation and damage to the mitochondrial structure. These changes are consistent with the activation of ferroptosis, as they are significantly exacerbated by the stimulation of Iron‐dextran. However, XNKQ acupuncture intervention effectively reverses these pathological changes. By means of ferroptosis‐related mechanisms such as restoring iron homeostasis, inhibiting lipid peroxidation, and protecting mitochondria, it simultaneously salvages the neuronal damage caused by 6 h rtPA thrombolysis and that aggravated by Iron‐dextran. This strongly demonstrates that ferroptosis is a key factor contributing to neuronal injury induced by delayed thrombolysis.

## 4 Discussion

In this study, we explored the impact of XNKQ acupuncture on delayed rtPA thrombolysis in rats with cerebral infarction and its association with ferroptosis. Our findings revealed that XNKQ acupuncture can ameliorate neurological deficits and improve blood flow perfusion in rats with cerebral ischemia and delayed rtPA treatment while also reducing infarct volume. Notably, XNKQ acupuncture significantly lowered the risk of HT and brain edema resulting from delayed rtPA thrombolysis, thereby enhancing the safety of rtPA treatment. Specifically, XNKQ acupuncture mitigated BBB disruption and neuronal damage by inhibiting ferroptosis. Furthermore, this inhibition of ferroptosis was achieved through the regulation of iron metabolism, modulation of lipid peroxidation levels, and preservation of mitochondrial structural integrity.

GV26 and PC6 are considered crucial acupoints in the XNKQ acupuncture protocol. According to traditional Chinese medicine theory, GV26 is a crucial emergency acupoint that facilitates the circulation of Qi and Blood. PC6 has a calming effect and is commonly used to treat symptoms such as palpitations, chest tightness, and insomnia. Research indicates that GV26 reduces the expression of angiotensin, which helps improve vascular function and enhances collateral circulation in the anterior cerebral circulation, alleviating cerebral ischemia (Dong et al. 2020). PC6 can significantly increase the cerebral oxygen supply and regulate autonomic nerve function. The combined use of GV26 and PC6 can produce synergistic effects, on the one hand, by promoting the circulation of qi and blood and improving vascular function to alleviate cerebral ischemia and, on the other hand, by regulating mental and autonomic nerve function to promote overall recovery (J. Li et al. 2014). To exclude the placebo effect of this acupuncture protocol and verify its therapeutic effect, we also designed a sham acupuncture group, following the protocol by Streitberger et al. (Streitberger et al. 2004; Takakura et al. 2015; Huang et al. 2013) Clinical evidence shows that XNKQ acupuncture significantly improves the clinical efficacy of rtPA intravenous thrombolysis in patients with cerebral infarction, reducing the incidence of symptomatic intracranial hemorrhage and the rate of adverse reactions (Y. Song, Zhang, et al. 2022). The earlier the acupuncture intervention is applied during the acute phase of cerebral infarction, the better the improvement in clinical symptoms (Peng et al. 2023). Hence, we chose the 6 h time point after the thrombus model establishment for acupuncture treatment in this study.

The BBB restricts the entry of certain substances from the blood into the brain, maintaining the basic stability of the internal environment of the brain tissue (Sifat et al. 2017). TJs are the main structures that constitute the BBB, and changes in the structure, distribution, and expression levels of TJ‐related proteins can lead to the opening or closing of TJs, thereby altering BBB permeability. After delayed rtPA thrombolysis in cerebral infarction, the protein expression of Claudin5 and ZO‐1, which are involved in the formation of TJs, is reduced (Nico and Ribatti 2012), and TEM images revealed that TJ structures are decomposed, the BBB structure is damaged and in a high‐permeability state, with increased permeability, and the degree of cerebral hemorrhage and edema continuously worsens. Our study revealed that after XNKQ acupuncture, the structure and functional integrity of the BBB in rats subjected to delayed rtPA thrombolysis were significantly improved, and the degree of HT and brain edema was reduced. In addition, BBB disruption is an important factor leading to neurological dysfunction and even neuronal cell death (Abbott et al. 2010). In the cerebral cortex of rats subjected to delayed rtPA thrombolysis, the number of neurons significantly decreased, and neuronal damage was observed morphologically. XNKQ acupuncture significantly reduced neuronal damage.

Iron is crucial in the transport of blood oxygen (Ward et al. 2014). Under acute ischemic conditions, the uptake of iron ions from the peripheral circulation by local brain tissue increases (Chen et al. 2011), with large amounts of Fe^3+^ entering the brain parenchyma through the highly permeable BBB, being reduced to free Fe^2+^ via TFR1 and stored in Ferritin (Słomka et al. 2015; Tan et al. 2016; DeGregorio‐Rocasolano et al. 2018). FPN1 is a transmembrane protein that can export excess Fe^2+^ from cells (Ortlund et al. 2005). Consequently, our findings revealed an increase in the protein expression of FPN1 and Ferritin, whereas the protein expression of TFR1 was decreased. GSH and GPX4 serve as quintessential markers of the antioxidant system. The membrane transporter SLC7A11 transports cystine from outside the cell into the cell, where it is further converted into cysteine, where it participates in the synthesis of GSH. GSH can block the formation of highly toxic hydroxyl radicals from Fe^2+^, thereby inhibiting ferroptosis. Disruption of the intake, storage, and excretion of iron ions in the brain can disrupt the balance of iron ions in the brain, leading to iron‐induced neuronal death (Aguirre et al. 2005). The disruption of cellular iron ion homeostasis leads to an increase in free Fe^2+^ in neurons. Excessive free Fe^2+^ reacts with mitochondria in the Fenton reaction, leading to an increase in reactive oxygen species, inhibiting the synthesis of GSH and the activity of GPX4 (Green 2019), resulting in impaired antioxidant systems, redox imbalance, and the accumulation of toxic lipid peroxidation products such as MDA, ultimately exacerbating neuronal ferroptosis (She et al. 2020). The colocalization of NeuN and Ferritin observed via immunofluorescence microscopy revealed that Ferritin accumulates in the brain parenchyma after cerebral infarction, causing neuronal damage. After acupuncture treatment, the colocalization of NeuN and Ferritin is reduced. Ferritin is an iron storage protein whose expression level is regulated by iron metabolism. Under iron overload conditions (induced by iron dextran), extracellular total ferritin in brain tissue may degrade and release iron ions (Fe^2+^), leading to a decrease in total protein levels in the tissue, while the degraded iron ions are transferred into cells and accumulate in neurons. Therefore, the decrease in total ferritin expression and the increase in intracellular iron deposition represent manifestations of different sites in the same pathological process, both reflecting iron metabolic disorders. Whether reducing iron deposition in neurons or restoring total ferritin expression, acupuncture has shown consistent effects in regulating iron metabolism and inhibiting ferroptosis. The colocalization of NeuN and Ferritin reveals the role of acupuncture in neuronal damage and ferroptosis, providing a different perspective for the treatment of AIS with acupuncture. Mitochondrial structural and functional damage is also a hallmark of ferroptosis. In the cerebral infarction model, many mitochondrial fragments were observed around the nuclei of neurons in the ischemic area, with ruptured outer mitochondrial membranes and reduced or absent cristae (G. Li et al. 2021). Tian et al. (2015) reported that acupuncture can improve the mitochondrial ultrastructure. Salvaging mitochondrial integrity and function can effectively prevent the occurrence of neuronal ferroptosis.

To reveal the specific mechanisms of ferroptosis in the effects of acupuncture intervention on delayed thrombolysis in the context of cerebral infarction, we added the ferroptosis inducer Iron‐dextran to the 6 h rtPA+Iron‐dextran group and the 6 h rtPA+Iron‐dextran+Acu group. Iron‐dextran is widely used in clinical and research processes and can establish an acute brain iron overload model in rats within a short time (Piloni et al. 2013). The data from this study indicate that after intervention with the ferroptosis inducer Iron‐dextran, the brain tissue Fe^2+^ levels in rats subjected to delayed thrombolysis are dysregulated, and the antioxidant stress capacity decreases, leading to the accumulation of lipid peroxidation and the occurrence of ferroptosis. This also explains why this group exhibited more severe neurological damage and BBB disruption. However, more importantly, the study reveals that XNKQ acupuncture exerts significant neuroprotective effects in the treatment of cerebral infarction following delayed rtPA thrombolysis through multi‐target and multi‐level comprehensive mechanisms. Specifically, in terms of neurological function improvement, acupuncture significantly reduces modified Bederson scores, decreases pathological turning frequency, and shortens adhesive removal time; TTC staining and laser speckle imaging confirm that acupuncture reduces cerebral infarct volume while increasing blood perfusion in ischemic areas. Regarding BBB protection, acupuncture operates through a triple mechanism‐molecularly upregulating Claudin5 and ZO‐1, ultrastructurally maintaining endothelial TJ integrity, and functionally reducing EB extravasation, hemoglobin content, and brain water content, ultimately lowering the risk of HT. In ferroptosis regulation, acupuncture improves iron metabolism by reducing brain tissue Fe^2+^ levels, decreasing Prussian blue positive deposits, and modulating iron metabolism‐related proteins, enhances antioxidant defense by elevating GSH levels, boosting GPX4 activity, and lowering MDA content; and preserves mitochondrial function by maintaining cristae integrity and reducing outer membrane rupture. These effects form a positive feedback loop—improving iron metabolism, alleviating oxidative stress, protecting mitochondria, reducing neuronal death, maintaining BBB stability, and preventing iron‐ion infiltration. Notably, acupuncture maintains its protective effects even after Iron dextran‐induced ferroptosis. These findings establish the potential of acupuncture as an adjunctive therapy to rtPA thrombolysis, addressing its critical limitation—the narrow therapeutic time window. By targeting the ferroptosis pathway, acupuncture may provide extended neuroprotection for patients beyond the conventional 4.5 h thrombolysis window (particularly in delayed treatment cases). However, the specific signaling pathways through which acupuncture regulates ferroptosis require further investigation. Future research should explore combination strategies with iron chelators or antioxidants to optimize therapeutic efficacy, ultimately providing a multi‐target treatment approach for acute cerebral infarction that integrates traditional medicine with modern neurobiology.

## 5 Conclusions

In conclusion, our findings demonstrate that XNKQ acupuncture can mitigate ferroptosis in neurons by modulating iron ion metabolism and reducing the accumulation of lipid peroxidation. In addition, it helps maintain the integrity of the BBB and alleviates complications caused by delayed rtPA thrombolysis in cerebral infarction, such as HT. Finally, XNKQ acupuncture can serve as an important and effective treatment during rtPA thrombolysis for AIS, enhancing the neuroprotective effects by improving the safety of thrombolysis therapy. These results provide a solid theoretical foundation for the clinical application of thrombolysis in treating AIS.

## Author Contributions

**Zheng Huang**: conceptualization, data curation, writing–original draft, writing–review and editing. **Tianliang Lu**: data curation, writing–original draft, writing–review and editing. **Xinyu Liu**: writing–review and editing, visualization. **Zhihui Zhang**: writing–review and editing, visualization. **Yangyang Song**: writing–review and editing, visualization. **Yiyang Li**: writing–review and editing, visualization. **Wentao Xu**: writing–review and editing, visualization. **Xinchang Zhang**: conceptualization, writing–review and editing, funding acquisition. **Guangxia Ni**: conceptualization, writing–review and editing, funding acquisition.

## Ethics Statement

The Institutional Animal Care and Use Committee of Nanjing University of Chinese Medicine (Approval No. 202404A014) approved the animal experiments conducted in this study, which were strictly in accordance with the guidelines established by the National Institutes of Health Animal Care and Use Committee.

## Conflicts of Interest

The authors declare no conflicts of interest.

## Peer Review

The peer review history for this article is available at https://publons.com/publon/10.1002/brb3.70644

## Data Availability

The data presented in this study are available on request from the corresponding author.
